# Ten-Year Death Trends Related to Dementia, Alzheimer’s Disease, and Epilepsy in England and Wales (2014–2023): A Descriptive Analysis of National Death Registration Data

**DOI:** 10.7759/cureus.93837

**Published:** 2025-10-04

**Authors:** Fatimot Disu, Basilia G Iwudibia, Olasunkanmi Olatunbosun

**Affiliations:** 1 Stroke/Neuroscience, University Hospital Southampton NHS Foundation Trust, Southampton, GBR; 2 Family Medicine, Phoenix Medical Clinic, St. Catharines, CAN; 3 Acute Medicine, Wye Valley NHS Trust, Hereford, GBR

**Keywords:** alzheimer’s disease, dementia, england and wales, epidemiology, epilepsy

## Abstract

Background: Dementia, including Alzheimer’s disease, and epilepsy are major neurological conditions contributing to mortality in England and Wales. Recent data suggest increasing dementia‑related deaths, particularly in ageing populations, while epilepsy remains a less frequent but significant cause of premature mortality.

Methods: This descriptive study analyzed national death registration data from the Office for National Statistics between 2014 and 2023. Deaths attributed to dementia (International Classification of Diseases, Tenth Revision (ICD-10) codes, F01, F03, G30) and epilepsy (ICD-10 codes, G40-G41) were included for individuals aged ≥20 years in England and Wales. Data were cleaned, filtered, and analyzed using Stata Statistical Software: Release 18 (StataCorp LLC., College Station, Texas, United States). Frequencies and percentages were calculated using frequency weights to reflect true death counts, and trends were visualized using line and bar graphs.

Results: A total of 622,965 deaths were recorded over the 10‑year period. Dementia and Alzheimer’s disease accounted for nearly all deaths (99.98%) as the recorded underlying (primary) cause of death on death certificates, predominantly among females (67.5%) and individuals aged ≥80 years (89.9%). Mortality increased from 2014 (7.7%) to 2018 (11.1%), declined in 2021 (9.2%) during the COVID‑19 pandemic, and rebounded in 2022 (10.5%) and 2023 (10.7%).

Conclusion: Dementia‑related mortality in England and Wales remains high and predominantly affects older women. Targeted prevention, improved care pathways, and continued monitoring of post‑pandemic trends are recommended.

## Introduction

Dementia, including that due to Alzheimer's disease, and epilepsy are the main neurological conditions that impose a great burden on the healthcare systems in England and Wales [1]. These disorders have also been implicated in the mortality rates more often in England and Wales, especially in the ageing population [2]. The World Health Organization (WHO) reported that dementia, such as Alzheimer's disease, has emerged as the sixth highest cause of death in the world [3]. In the United Kingdom, both dementia and Alzheimer's have regularly been in the list of the leading causes of death, beating most other chronic ailments [4]. Similarly, epilepsy, although lower in prevalence, has a high risk of early mortality, and contributes towards premature death, sudden unexpected death in epilepsy (SUDEP) related to status epilepticus, drowning, and seizure-related injuries, especially in the vulnerable population group [5]. 

Dementia is a spectrum of progressive neurological disorders that are marked by the deterioration of cognitive abilities to the extent that the person finds it difficult to perform their day-to-day activities [6,7]. The most common forms of dementia include Alzheimer's disease (International Classification of Diseases, Tenth Revision (ICD-10) code, G30), vascular dementia (ICD-10 code, F01), and unspecified dementia (ICD-10 code, F03) [8]. The geriatric population suffers disproportionately due to these conditions, and they are also associated with a higher rate of dependence, hospitalizations, and, finally, death [9]. Due to both population ageing and evidence of rising dementia incidence in recent years (with changes in risk-factor profiles and diagnostic ascertainment proposed as contributors), deaths caused by dementia have shown an increasing trend in England and Wales [10]. 

Epilepsy is a neurological disorder classified under ICD-10 codes G40 and G41, which is chronic in nature and is characterized by recurrent seizures [11]. The people who die in relation to epilepsy most likely do so at an earlier age than those who succumb to dementia, although epilepsy is most common among children and elders [12]. Mortality in epilepsy has numerous causes and might involve injuries that occur during the seizure and status epilepticus and complications of comorbid mental or physical health conditions [13]. Even though epilepsy is not on the list of leading causes of death, it is a leading underlying cause of premature death and years of life lost, rendering the condition a key priority issue among neurologists and other health professionals in the field of public health [14]. 

The COVID-19 pandemic made death rates related to neurological disorders even more complex [15]. During the COVID-19 pandemic (2020-2022), the number of deaths associated with dementia soared, in part because of the susceptibility of long-term care home residents and the loss of continuity of chronic illness management [16]. With the country leaving the pandemic, 2023 was a significant year to explore whether mortality tendencies linked with dementia, Alzheimer's disease, and epilepsy reverted to the pre-pandemic trends or whether it was a long-term change [17]. 

The Office for National Statistics (ONS) provides detailed annual data on causes of death, broken down by ICD-10 codes used in England and Wales [18]. ONS death registration data from 2014 to 2023 provides a recent, clear, and current picture of the death burden of neurological conditions broken down by age, sex, and region [19-21]. This study aims to describe a 10-year trend in the annual number of deaths attributed to dementia (ICD-10 codes, F01, F03, G30) and epilepsy (ICD-10 codes, G40-G41) in England and Wales using publicly available 2014-2023 death registration data. 

## Materials and methods

Study design and data source 

This was a descriptive epidemiological study analyzing mortality data from 2014 to 2023 in England and Wales [21]. Data were extracted from the ONS death registration database, which records all legally registered deaths within these countries. Each record includes demographic information (age, sex, and place of residence) and causes of death coded using the ICD‑10.

Study population and eligibility criteria 

The study population comprised individuals aged 20 years and older whose deaths were registered in England or Wales during the study period. The lower age limit of 20 years was chosen due to the rarity of dementia and epilepsy-related deaths in children and adolescents, ensuring clinical and epidemiological relevance. Age groups were categorized as 20-34, 35-49, 50-64, 65-79, and 80 years and over. The study included deaths in which any one of the ICD-10 codes F01, F03, or G30 (dementia/Alzheimer’s) or G40-G41 (epilepsy) was recorded as the underlying (primary) cause of death on the death certificate.

Records where these codes appeared only as contributing (secondary) causes were not included in the primary analyses. Additionally, deaths recorded under the category “England, Wales and Non-residents” were excluded to maintain focus on resident populations. Records with missing or indeterminate sex were also excluded. “All people” summary entries, which appeared as aggregated categories in the dataset, were omitted to avoid duplication. 

Variables of interest 

Key variables included year of registration, sex (male or female), age group, and underlying cause of death based on ICD‑10 coding. Dementia and Alzheimer’s disease were grouped as a single category, while epilepsy was analyzed separately. Both absolute counts and proportions of deaths were assessed. 

Data management and statistical analysis 

Data cleaning and analysis were conducted using Stata Statistical Software: Release 18 (StataCorp LLC., College Station, Texas, United States). The dataset was filtered to include only deaths meeting the study inclusion criteria, and age and sex variables were recoded into standardized categories. Because the dataset consisted of aggregated death counts (each row representing the number of deaths for a specific year, age group, sex, and cause), analyses were performed using frequency weights to ensure that percentages and totals accurately reflected the underlying counts. Descriptive statistics, including frequencies and weighted percentages, were generated to summarize death by year, sex, and age group. Temporal trends were illustrated using line graphs, while age- and sex-specific distributions were presented using grouped bar charts. No inferential statistical tests were conducted, as the study objective was purely descriptive. 

Ethical considerations 

This study utilized publicly available, anonymized mortality data from the ONS. As the data contain no personal identifiers and are in the public domain, formal ethical approval and informed consent were not required. The study was conducted in compliance with the principles of good research practice and adhered to applicable data protection and confidentiality standards. 

## Results

Table 1 summarizes the annual number of deaths attributable to dementia, Alzheimer’s disease, and epilepsy among individuals aged ≥20 years in England and Wales over a 10‑year period from 2014 to 2023. A total of 622,965 deaths were attributed to dementia, Alzheimer’s disease, and epilepsy in England and Wales during the 10‑year study period from 2014 to 2023. The temporal distribution of these deaths revealed progressive increases in annual death counts during the early years of observation, rising from 48,072 deaths (7.7%) in 2014 to a peak of 69,158 deaths (11.1%) in 2018. Following the 2018 peak, annual death counts remained relatively stable until a notable decrease in 2021, when deaths dropped to 57,268 (9.2%). In the post-pandemic period, death counts rebounded, rising to 65,620 deaths (10.5%) in 2022 and 66,558 deaths (10.7%) in 2023, suggesting a return toward pre‑pandemic levels. 

**Table 1:** Annual deaths from dementia, Alzheimer’s disease, and epilepsy in England and Wales, 2014–2023 Annual death figures represent combined deaths from dementia (F01, F03, G30) and epilepsy (G40–G41) in individuals aged ≥20 years. Analyses were performed using frequency weights, ensuring that percentages reflect the true number of deaths rather than the number of aggregated rows in the dataset.

Year	Deaths due to dementia/Alzheimer’s/epilepsy	Percentage of total deaths due to dementia/Alzheimer's/epilepsy (n=622,965)
2014	48,072	7.7%
2015	58,042	9.3%
2016	59,152	9.5%
2017	67,339	10.8%
2018	69,158	11.1%
2019	66,136	10.6%
2020	65,620	10.5%
2021	57,268	9.2%
2022	65,620	10.5%
2023	66,558	10.7%

Table 2 presents the distribution of deaths due to dementia, Alzheimer’s disease, and epilepsy by sex and age group. Across the study period, female deaths predominated, accounting for 420,330 (67.5%) of total deaths, compared to 202,635 (32.5%) in males. Almost all deaths in both sexes were attributed to dementia and Alzheimer’s disease, with only 123 epilepsy-related deaths recorded, all occurring among females. 

**Table 2:** Deaths by sex and age group for dementia/Alzheimer’s disease and epilepsy, England and Wales, 2014–2023 Deaths represent combined cases from dementia (F01, F03, G30) and epilepsy (G40–G41) among individuals aged ≥20 years. Frequencies and percentages were computed using frequency weights to reflect true death counts rather than row totals from the aggregated dataset.

Variable (including Alzheimer’s)	Dementia/Alzheimer’s disease (n=622,842), n (%)	Epilepsy (n=123), n (%)	Total (n=622,965), n (%)
Sex			
Female	420,207 (67.47%)	123 (100%)	420,330 (67.47%)
Male	202,635 (32.53%)	0 (0.0%)	202,635 (32.53%)
Age group			
20–34	0 (0%)	123 (100%)	123 (0.02%)
35–49	0 (0%)	0 (0%)	0 (0.00%)
50–64	0 (0%)	0 (0%)	0 (0.00%)
65–79	63,124 (10.13%)	0 (0%)	63,124 (10.13%)
80+	559,718 (89.87%)	0 (0%)	559,718 (89.85%)

**Figure 1:**
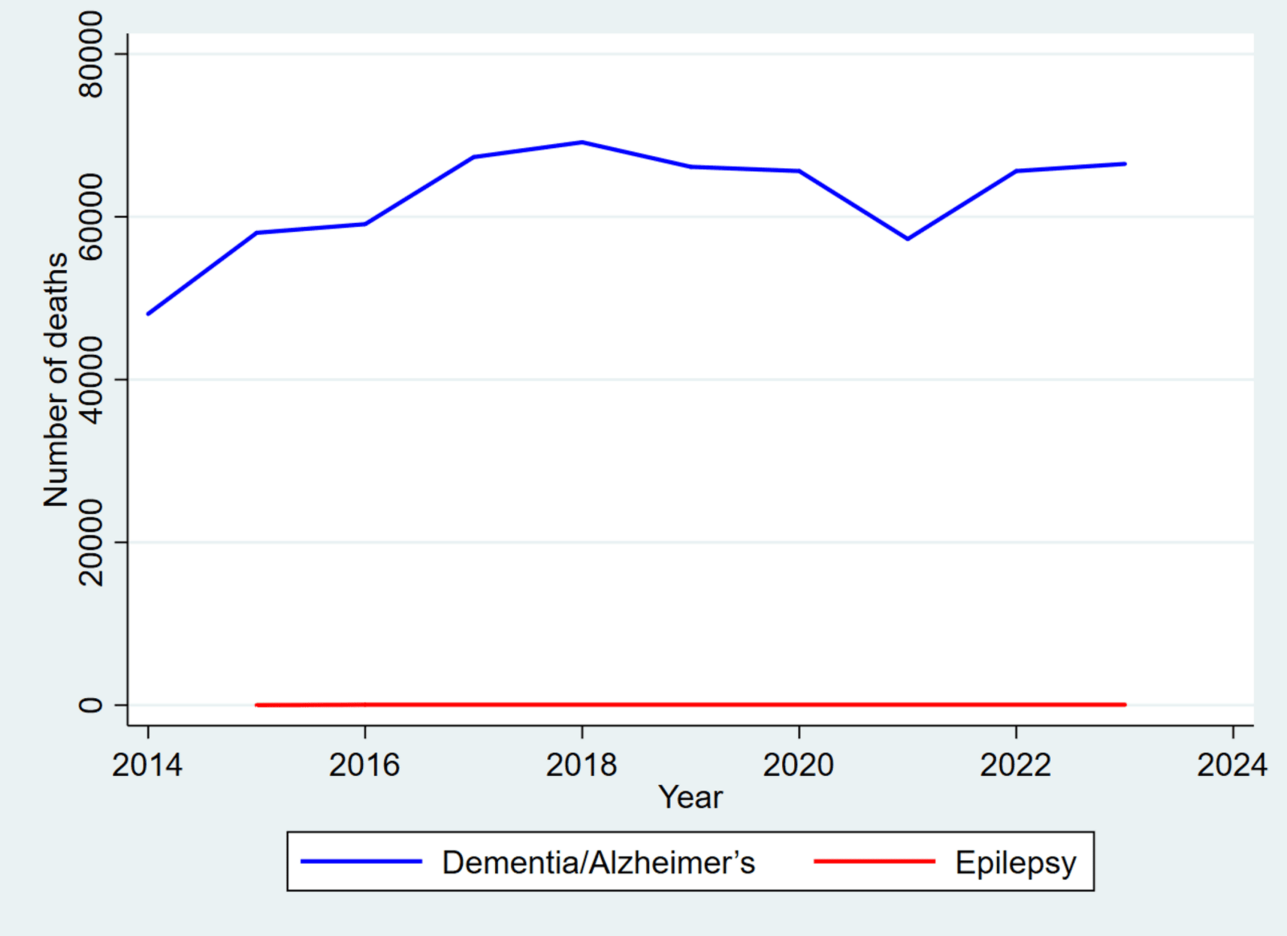
Annual death trends in dementia/Alzheimer’s disease and epilepsy, England and Wales (2014–2023) This line graph displays the annual number of deaths attributed to dementia and Alzheimer’s disease (ICD-10 codes F01, F03, G30) versus epilepsy (G40–G41) from 2014 to 2023. Mortality counts were calculated using frequency weights from national death registration data.

Figure 1 visually illustrates the death trends associated with deaths from dementia, Alzheimer’s disease, and epilepsy in England and Wales from 2014 to 2023. This graphic complements the tabulated data and provides deeper insight into temporal shifts and subgroup differences over the study period. 

In extreme contrast, epilepsy-related deaths remained exceedingly low throughout the decade and are almost indistinguishable on the graph due to their minimal contribution to overall neurological mortality. 

## Discussion

This study analyzed 10‑year national trends in the annual number of deaths for dementia, Alzheimer’s disease, and epilepsy in England and Wales, drawing on death registration data from 2014 to 2023. The findings revealed several important patterns. First, dementia and Alzheimer’s disease together accounted for the overwhelming majority of neurological deaths, while epilepsy contributed a minimal proportion. Second, mortality from dementia and Alzheimer’s disease rose steadily between 2014 and 2018, stabilized thereafter, and declined briefly during the COVID‑19 pandemic period before rebounding in subsequent years. Third, mortality was heavily concentrated among older adults, particularly those aged 80 years and above, and was markedly higher among females compared to males. In contrast, epilepsy‑related deaths were rare and occurred exclusively in younger adults aged 20-34 years. Possible explanations for epilepsy deaths being concentrated in younger adults include higher relative incidence of uncontrolled epilepsy in younger age groups, increased risk of SUDEP among younger and poorly controlled cases, seizure-related accidents, and fewer competing causes of death that would otherwise reduce the proportionate representation of epilepsy deaths in older age groups; the small number of epilepsy deaths in our dataset limits confirm conclusions. These data highlight pronounced demographic differences: the disproportionate burden of dementia-related deaths among older adults and the minimal overall contribution of epilepsy to the total death burden during the study period.

The upward trajectory in dementia‑related deaths observed between 2014 and 2018 aligns with previously reported trends in England and Wales, which have documented rising dementia mortality over the past two decades [1,2]. This increase has been attributed to an ageing population, improved disease recognition, and changes in certification practices that emphasize dementia as an underlying cause of death [10,18]. The disproportionate burden among older adults is consistent with the natural history of dementia, as prevalence and mortality increase exponentially with advancing age [6,9]. The marked female predominance mirrors findings from prior United Kingdom and international studies [4,9], reflecting both women’s longer life expectancy and evidence suggesting sex‑specific biological and hormonal risk factors for Alzheimer’s pathology [4]. This female predominance likely reflects both women’s longer life expectancy and documented sex-specific differences in dementia prevalence and survival. This observed rise may reflect both the growing prevalence of dementia in ageing populations and improvements in diagnostic ascertainment and death-certification practices for neurodegenerative disorders during that period. Overall, the observed fluctuations likely reflect a complex interplay of population ageing, changing disease epidemiology, evolving diagnostic and certification practices, and external public health events (notably the COVID-19 pandemic) that affected both disease incidence and death reporting. 

The momentary decline in mortality during 2021 coincides with the COVID‑19 pandemic, a period characterized by widespread disruptions to healthcare services and competing mortality risks. Several studies have reported excess deaths among dementia patients during the pandemic, particularly in care home residents [15-17]. However, variations in death certification and shifts in prioritizing COVID‑19 as the primary cause of death may have temporarily altered reported dementia mortality [16]. The rebound observed in 2022-2023 suggests either a return to pre‑pandemic trends or potential delayed mortality effects related to pandemic‑era health service disruptions. This reduction coincided with the height of the COVID-19 pandemic, a period characterized by substantial changes in mortality patterns nationally, including high excess mortality attributable to COVID-19 and the potential for under-ascertainment or reclassification of other underlying causes of death.

Epilepsy accounted for only 123 deaths over the decade, all occurring in young adults. Although epilepsy mortality is considerably lower than dementia mortality in absolute numbers, previous research highlights that deaths in epilepsy often occur prematurely and may involve SUDEP or seizure‑related accidents [5,12,13]. The absence of epilepsy deaths among older age groups in this dataset may reflect both lower prevalence of active epilepsy in late life and competing risks from other comorbidities. These contrasting patterns emphasize the disproportionate burden of dementia compared to epilepsy in the adult population. 

Strengths and limitations 

A key strength of this study is its use of comprehensive, population‑level mortality data from the ONS, ensuring near‑complete capture of deaths and standardized coding across a 10‑year span [19]. The descriptive design enabled detailed characterization of trends by year, sex, and age group, offering an updated overview of mortality patterns for two major neurological conditions. 

However, several limitations warrant consideration. The analysis was restricted to underlying cause of death coding; therefore, deaths in which dementia or epilepsy were contributing but not primary factors may be underestimated. Lack of individual‑level data on comorbidities, socioeconomic status, or ethnicity precluded more nuanced stratification of mortality risks. As a descriptive study, causal inferences cannot be drawn, and the findings should be interpreted in the context of broader epidemiological and health service changes over the study period. Another limitation of this study is the sole reliance on death registration data, without comparison to the number of individuals diagnosed with dementia or epilepsy during the study period. This limitation restricts the ability to calculate disease-specific mortality rates or assess mortality relative to prevalence. Consequently, the findings should be interpreted as patterns in the absolute number of deaths rather than mortality risks within the affected populations.

Future research should incorporate linked longitudinal datasets that combine mortality records with hospital, primary care, and social care data to better characterize multimorbidity, care pathways, and socioeconomic inequalities. Further exploration of dementia subtypes, epilepsy phenotypes, and their distinct mortality trajectories could inform targeted prevention and management strategies. Additionally, examining post‑pandemic mortality trends beyond 2023 will clarify whether observed rebounds reflect a return to prior trajectories or ongoing structural shifts in neurological disease burden. 

## Conclusions

Death from dementia and Alzheimer’s disease in England and Wales remains substantial and has followed an overall upward trajectory over the past decade, with temporary concern during the COVID‑19 pandemic. The burden falls disproportionately on older adults and women, while epilepsy‑related deaths remain rare but concentrated among younger individuals. These findings reinforce the need for continued public health focus on dementia prevention and management, while also addressing targeted epilepsy mortality reduction strategies in younger populations. 
